# Giant adrenal pseudocyst with unclear preoperative origin: a case report and literature review

**DOI:** 10.3389/fsurg.2026.1883619

**Published:** 2026-09-03

**Authors:** Shouyi Long, Bin Xiong, Zhen Long, Bo Li, Xiujun Yuan, Shulian Chen

**Affiliations:** 1 Department of Urology, Renhuai Traditional Chinese Medicine Hospital, Renhuai, Guizhou, China; 2 Department of Urology, Affiliated Hospital of Zunyi Medical University, Zunyi, Guizhou, China

**Keywords:** adrenal incidentaloma, dense adhesions, diagnostic difficulty, differential diagnosis, giant adrenal pseudocyst, retroperitoneal cystic mass

## Abstract

**Introduction:**

Adrenal cysts are relatively rare, and most are nonfunctional and lack specific clinical manifestations, often being discovered incidentally during imaging examinations. For giant adrenal cysts with a maximum diameter exceeding 10 cm, especially when accompanied by degenerative changes such as hemorrhage, fibrosis, and calcification, compression of adjacent organs and obscuration of anatomical planes may increase the difficulty of determining the origin preoperatively, and they may even be misdiagnosed as pancreatic, renal, or other retroperitoneal cystic lesions. This article reports one case of a giant adrenal pseudocyst with unclear origin before surgery and discusses its diagnostic and surgical management strategies.

**Case report:**

A 36-year-old woman was admitted after a left retroperitoneal mass was incidentally discovered during trauma evaluation one month earlier. Contrast-enhanced CT showed a cystic low-density lesion measuring approximately 108 mm × 102 mm × 108 mm in the left retroperitoneal space, with marginal calcification. The left adrenal gland was not distinctly identified, and the lesion was closely related to the stomach, spleen, and pancreas. The organ of origin remained uncertain preoperatively, with cystic lesions of adrenal, pancreatic, or gastric origin considered in the differential diagnosis. After multidisciplinary discussion, laparoscopic resection was performed. Intraoperatively, the lesion was densely adherent to surrounding organs and its origin was difficult to determine; resection was completed after decompression, and the residual cyst wall on the splenic side was treated with electrocautery. Postoperative pathology confirmed a left adrenal pseudocyst with calcification. The patient recovered smoothly, and no complications were observed during follow-up.

**Conclusion:**

Giant complex adrenal pseudocysts may mimic other left retroperitoneal cystic lesions, and the diagnostic difficulty lies in determining the organ of origin. For giant retroperitoneal cystic lesions of unclear origin, adrenal origin should be included as an important differential diagnosis, and comprehensive decisions should be made based on imaging, endocrine evaluation, and multidisciplinary discussion; if no safe dissection plane exists intraoperatively, individualized management should be implemented.

## Introduction

Adrenal cysts are relatively rare, accounting for only about 1%–2% of adrenal incidentalomas in clinical practice, while autopsy studies have shown a prevalence of approximately 0.06%–0.18% (1). Because most adrenal cysts are nonfunctional and lack specific clinical manifestations, they are often discovered incidentally during imaging examinations performed for other diseases or routine health evaluations (2). According to histopathological characteristics, adrenal cysts are traditionally classified into pseudocysts, endothelial cysts, epithelial cysts, and parasitic cysts, among which pseudocysts are more common in clinical and surgical series (3).

In case reports and related reviews, adrenal cysts with a maximum diameter exceeding 10 cm are usually classified as giant adrenal cysts (4, 5). Unlike simple thin-walled cysts, giant or complex adrenal cysts may be accompanied by degenerative changes such as intracystic hemorrhage, fibrosis, and calcification. As the lesion enlarges, normal adrenal tissue may become thinned and difficult to identify due to compression, while exerting significant mass effect on adjacent organs, thereby increasing the difficulty of determining the organ of origin preoperatively (6–8). Previous case reports and reviews have documented that such lesions may be misdiagnosed or provisionally diagnosed as pancreatic cystic tumors, cystic renal cell carcinoma, hepatic cysts, and other lesions (9–12). Therefore, failure to recognize a possible adrenal origin may not only complicate the preoperative differential diagnosis but may also result in omission of the adrenal-specific hormonal assessment and multidisciplinary evaluation recommended for indeterminate adrenal masses (13).

Based on this, this article reports a case of a giant adrenal cyst whose origin could not be determined preoperatively or intraoperatively, and which was confirmed postoperatively by pathology as a pseudocyst type. Combined with this case report and related literature review, we focused on discussing the diagnostic dilemma of giant adrenal pseudocysts, intraoperative management strategies when relationships with surrounding organs are complex, and their clinical implications.

## Case report

The patient was a 36-year-old woman who was admitted for “incidentally discovered left retroperitoneal mass during trauma examination for 1 month.” The patient had no symptoms suggestive of catecholamine excess, such as headache, palpitations, or hyperhidrosis. No overt clinical features suggestive of cortisol excess were identified, and she had no history of hypertension. No symptoms related to mass effect or infection were reported. Her past medical history was unremarkable. There was no known family history of adrenal tumors or hereditary tumor syndromes, including multiple endocrine neoplasia (MEN) and von Hippel–Lindau (VHL) disease, and no relevant psychosocial history was identified. Physical examination revealed no obvious positive findings.

**Figure 1:**
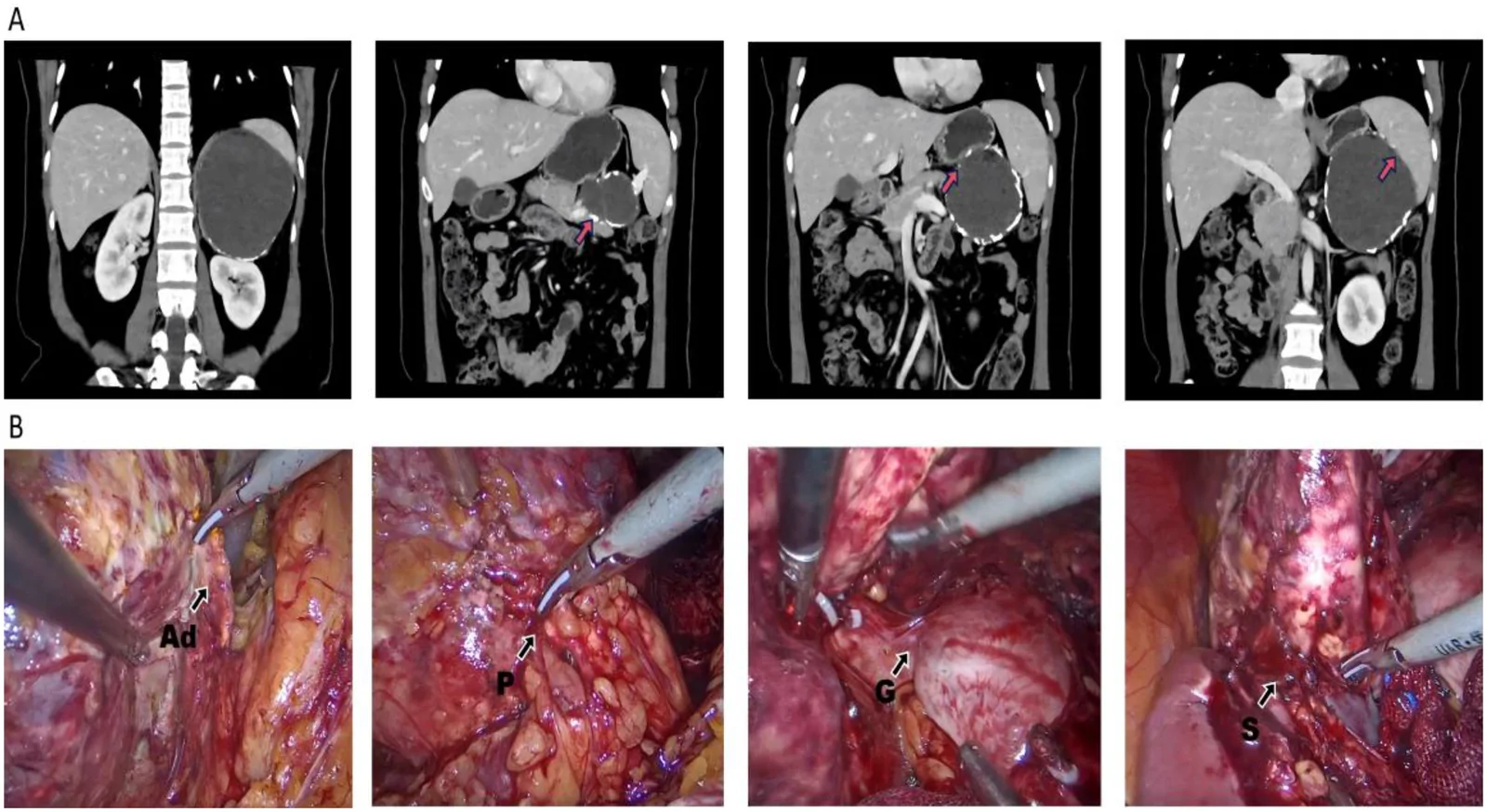
**(A)** Contrast-enhanced abdominal CT showed that the left adrenal gland was not distinctly identified. A giant cystic low-density lesion was seen in the left retroperitoneum, closely related to the stomach, spleen, and pancreas, with compression and displacement of the left kidney and no obvious enhancement on contrast-enhanced imaging. The arrows in the second, third, and fourth images indicate the interfaces between the lesion and the pancreas, stomach, and spleen, respectively. **(B)** Intraoperative images showing the adhesions between the giant left retroperitoneal cyst and adjacent organs. From left to right, Ad, adrenal gland; P, pancreas; G, stomach; and S, spleen. The arrows indicate the sites of adhesion between the cyst and the corresponding adjacent organs.

Contrast-enhanced abdominal CT showed that the left adrenal gland was not distinctly identified, and a cystic low-density lesion measuring approximately 108 mm × 102 mm × 108 mm was observed in the left retroperitoneal space. Arc-shaped and punctate calcifications were seen at the lesion margin. The lesion was closely related to the stomach, spleen, and pancreas, with compression and displacement of the left kidney. No obvious enhancement was observed on contrast-enhanced scanning (Figure 1A). Ultrasonography demonstrated a cystic-solid mass of mixed echogenicity between the upper pole of the left kidney and the spleen. Gastroscopy showed no obvious abnormalities. Preoperative endocrine-related tests showed: supine renin 8.29 μIU/mL, aldosterone 22.50 pg/mL, aldosterone/renin ratio 2.71; upright renin 8.75 μIU/mL, aldosterone 21.70 pg/mL, aldosterone/renin ratio 2.48. Renin and aldosterone were measured because an adrenal origin could not be excluded preoperatively, although the patient was normotensive. Plasma free or urinary fractionated metanephrines and cortisol assessment, including a 1-mg overnight dexamethasone suppression test, were not performed preoperatively. Potassium, sodium, chloride, and serum and urine amylase showed no obvious abnormalities. Based on imaging and laboratory findings, a giant cystic lesion in the left retroperitoneum was considered.

**Figure 2:**
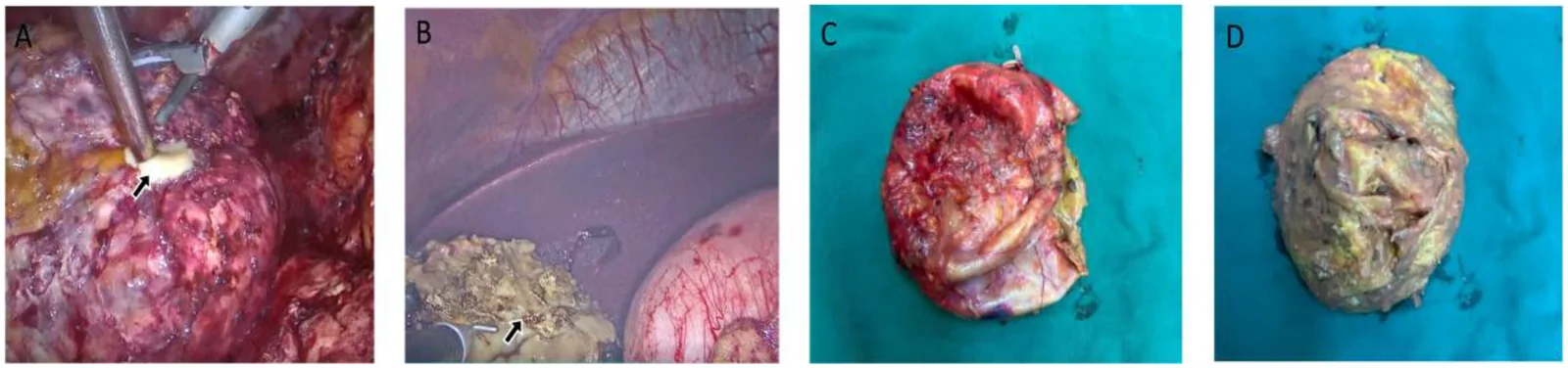
**(A)** After decompression of the giant left retroperitoneal cyst, milky cystic fluid was seen flowing out. **(B)** Electrocautery ablation was performed on the residual cyst wall closely adherent to the spleen to destroy the cyst wall structure. **(C)** Gross appearance of the resected specimen. **(D)** The cut specimen showed a leather-like appearance with thickened cyst wall and unilocular cystic structure. The cyst contained milky viscous fluid, and yellow calcified foci were observed on the inner wall.

After multidisciplinary discussion and full communication with the patient regarding the treatment plan, a transabdominal laparoscopic resection of a left retroperitoneal tumor was performed. Intraoperatively, a cystic mass with a diameter of approximately 11.0 cm was found in the left retroperitoneal space. The mass showed dense adhesions to the left adrenal gland, posterior gastric wall, and pancreas, with no clear boundary with the spleen, and its organ origin could still not be determined intraoperatively (Figure 1B). Considering the large size of the lesion, which affected exposure and dissection, the cyst wall was incised intraoperatively for decompression, and milky cystic fluid was aspirated (Figure 2A). The cyst fluid amylase was measured to exclude pancreatic origin, and the result was normal. Due to the absence of a clear anatomical plane between the lesion and the spleen, a portion of the cyst wall closely adherent to the spleen was preserved and treated with electrocautery (Figure 2B).

After surgery, the cut specimen showed a leather-like mass with thickened cyst wall and a unilocular cystic structure. The gross appearance of the resected specimen is shown in Figure 2C. On sectioning, the cyst contained milky white viscous fluid, and yellow calcified foci were observed on the inner wall of the cyst (Figure 2D). Postoperative pathological examination indicated cystic degeneration of the tissue, fibrous proliferation of the cyst wall, partial adrenal cortical structures in some areas, calcification formation, cholesterol crystals within the cavity, and no obvious cellular atypia. The final pathological diagnosis was an adrenal pseudocyst with calcification. The patient recovered smoothly after surgery, the drainage tube was removed on postoperative day 4, and the patient was discharged on postoperative day 5. At the 3-month postoperative follow-up, no surgery-related complications were observed.

## Discussion

For a large cystic mass in the left upper retroperitoneum, preoperative evaluation should first focus on determining the organ of origin (14). Cross-sectional imaging should assess whether the ipsilateral adrenal gland can be identified separately and whether the lesion is continuous with adjacent organs, particularly the pancreas, kidney, stomach, or spleen. The pattern of displacement of surrounding structures may provide additional clues to the site of origin. The lesion should then be characterized according to internal enhancement, wall features, septations, mural nodules, hemorrhage, and calcification. If the organ of origin remains uncertain, additional imaging should be selected according to the leading differential diagnosis. When an adrenal origin remains possible, appropriate hormonal evaluation and multidisciplinary assessment should be incorporated before intervention (13). Representative previously reported cases of giant adrenal pseudocysts are summarized in Table 1. These cases further demonstrate that the organ of origin may remain uncertain preoperatively or may be misattributed to adjacent organs, such as the pancreas, kidney, or liver.

**Table 1:** Representative published cases of giant adrenal pseudocysts.

Study	Patient demographics/lesion size	Preoperative diagnosis	Surgical approach	Pathological subtype/findings	Outcome
Mohan et al. (15)	16/F; R, 11 × 11 × 10 cm	Adrenal cystic lesion; malignancy suspected	Open *en bloc* excision with ipsilateral nephrectomy	Adrenal pseudocyst; fibrous wall with calcification and hemosiderin-laden macrophages	NR
Demir et al. (16)	45/F; L, 13 × 5 × 9 cm	Upper-pole renal cyst	Retroperitoneoscopic marsupialization	Adrenal pseudocyst; hyalinized and calcified wall	NR
Ujam et al. (17)	39/F; R, 29 × 20 × 17 cm	Upper-pole renal cyst	Laparoscopic adrenalectomy	Hemorrhagic adrenal pseudocyst	Asymptomatic at 6 months
Passoni et al. (18)	69/M; L, 12 × 9 × 7 cm	Adrenal carcinoma suspected	Open adrenalectomy with splenectomy and pancreatic tail resection	Adrenal pseudocyst with calcification and cholesterol crystals	Uneventful recovery; discharged on POD 12
Patnaik et al. (11)	50/M; R, 21 × 18 × 15 cm	Hepatic cyst	Open surgical excision	Hemorrhagic adrenal pseudocyst	Postoperative seroma; presenting symptoms resolved
Paramythiotis et al. (19)	28/F; L, 11.7 × 9.3 × 6.6 cm	Adrenal vs. pancreatic lesion	Open surgical excision	Adrenal pseudocyst	No abnormalities at 3-month follow-up
Shettar et al. (20)	40s/F; L, 13 × 12 × 11 cm	Large adrenal cyst; malignancy considered	Laparoscopic cyst excision	Adrenal pseudocyst; no malignancy	Asymptomatic at 6 months
Bobkiewicz et al. (21)	52/M; R, 23 × 16 cm	Adrenal cyst favored	Open adrenalectomy after cyst decompression	Hemorrhagic adrenal pseudocyst	Uneventful recovery; discharged on POD 5

F, female; M, male; L, left; R, right; NR, not reported; POD, postoperative day.

From an imaging perspective, giant or complex adrenal cystic lesions are not completely devoid of CT clues. Previous studies have shown that adrenal pseudocysts may present as purely cystic, cystic-solid, or even predominantly solid lesions. Among the more suggestive features are the absence of obvious intracavitary enhancement and the relatively common presence of mural or central calcification (22, 23). In this case, the combination of “unclear visualization of the normal left adrenal gland, arc-shaped and punctate calcifications at the lesion margin, no obvious enhancement on contrast-enhanced imaging, and compression and displacement of surrounding organs” is actually more consistent with the imaging features of giant complex adrenal cystic lesions. In comparison, the differential diagnosis of pancreatic cystic lesions should focus more on whether the lesion is continuous with the pancreatic parenchyma, whether there is communication with the pancreatic duct, whether the main pancreatic duct is dilated, and whether there are mural nodules, septations, or cyst wall enhancement. MRI/MRCP provides additional value in demonstrating ductal communication and complex intracystic structures, while EUS helps further evaluate mural nodules and other high-risk features. However, it should be noted that the absence of definite pancreatic duct communication cannot completely exclude pancreatic-origin lesions (24, 25). In the present case, MRI/MRCP was not performed because the preoperative diagnostic work-up proceeded directly from contrast-enhanced CT to surgical planning. As MRI/MRCP might have facilitated assessment of the lesion's relationship with the pancreas and pancreatic duct, its absence represents a limitation of the preoperative diagnostic evaluation. Gastric-origin lesions are relatively rare, but gastric duplication cysts can indeed mimic left adrenal cysts, and routine gastroscopy may not necessarily reveal specific abnormalities (26). Therefore, when encountering a giant cystic mass in the left upper abdomen, imaging evaluation should focus on the relationship with surrounding tissues and organs.

In addition, for retroperitoneal cystic lesions suspected to originate from the adrenal gland, preoperative evaluation should not be limited to morphological assessment alone, but should also incorporate relevant hormonal evaluation for comprehensive analysis. The 2023 ESE guidelines emphasize that all adrenal incidentalomas should undergo basic hormonal evaluation. For example, in principle, a dexamethasone suppression test should be performed. For lesions not consistent with typical benign adenoma imaging features, plasma free metanephrines or urinary fractionated metanephrines should also be measured. In patients with hypertension or unexplained hypokalemia, the aldosterone/renin ratio should be further assessed (13). In this case, preoperative ARR and basic laboratory tests were completed; however, metanephrine testing was not performed preoperatively. Although the patient lacked typical symptoms of catecholamine excess, this cannot fully replace the corresponding biochemical testing. Metanephrine testing and assessment for cortisol excess, including a 1-mg overnight dexamethasone suppression test, were not performed preoperatively. Therefore, the preoperative biochemical evaluation was incomplete and could not reliably exclude pheochromocytoma or autonomous cortisol secretion. In this case, severe adhesions between the cyst and surrounding tissues and organs were observed intraoperatively. Although there is no unified mechanism in the current literature, a relatively reasonable analytical framework has been proposed. Goel et al. reported in a series of cystic adrenal lesions that for larger lesions, the reason for dense adhesions with adjacent structures remains unclear, but may be related to leakage of cyst fluid beyond the capsule causing a surrounding tissue reaction and adhesion formation (6). On the other hand, recent systematic reviews have also indicated that adrenal pseudocysts are often accompanied by degenerative changes such as old hemorrhage, fibrosis, and calcification (27). Combined with the fact that pathology confirmed a pseudocyst type, preoperative imaging showed calcification, and postoperative findings revealed yellow calcified foci within the cyst with milky viscous contents, the severe adhesions in this case were likely not solely attributable to lesion enlargement, but more likely related to chronic degeneration after long-term existence of the lesion, old hemorrhage, or secondary fibrotic repair following leakage of cyst contents.

Although adrenal cysts do not always require surgical treatment, current studies generally suggest that surgical intervention should be preferred when the lesion is large, has significant mass effect, presents with complex imaging features or unclear origin, cannot reliably exclude malignancy, or has hormonal secretory function (2, 28, 29). The reason is that as the lesion increases in size, surgical complexity and perioperative risk also increase, which may delay postoperative recovery. This case was also similar; given the features of a large lesion, complex imaging appearance with unclear origin, and obvious mass effect on surrounding organs, we ultimately decided to perform surgical resection rather than sclerotherapy, as definitive histopathological diagnosis was required. The intraoperative management in this case is also of practical significance. For example, for giant left upper abdominal cystic lesions with unclear origin adjacent to important organs, a transabdominal laparoscopic approach can provide a larger working space, more familiar anatomical landmarks, and a wider operative field, thereby facilitating intraoperative assessment of the anatomical relationship between the lesion and surrounding structures (30, 31). In addition, we suggest that for very large cystic lesions, when they affect intraoperative manipulation, decompression can improve exposure and reduce the difficulty of subsequent dissection. Previous cases of giant adrenal cysts have shown that decompression followed by dissection and resection is one of the feasible minimally invasive strategies under specific conditions (32, 33). In addition, this case had a close relationship with the pancreas. Intraoperatively, fenestration and aspiration of milky cystic fluid were performed, and timely measurement of cyst fluid amylase helped exclude a pancreatic origin. Furthermore, in this case there was no clear anatomical boundary on the splenic side, and forced dissection carried a risk of splenic injury and massive bleeding. Therefore, a small portion of the cyst wall densely adherent to the spleen was retained and treated with electrocautery ablation. This represented an individualized balance between maximal lesion removal and patient safety.

Nevertheless, the potential risk of recurrence after partial cyst wall excision should be considered. Neri and Nance reported fluid reaccumulation in 32% of benign adrenal cysts treated by aspiration alone (34). In contrast, Castillo et al. reported no imaging-detected recurrence in eight surgically treated patients at a mean follow-up of 18.5 months (35), and El-Hefnawy et al. reported no recurrence after surgical treatment during a mean follow-up of 90 months (36). However, these findings cannot be directly extrapolated to the present approach, and specific evidence regarding electrocautery ablation of residual adrenal pseudocyst wall is lacking. Given this uncertainty, we plan to perform abdominal ultrasonography every 6 months for at least 3 years to monitor for cyst reaccumulation, with additional contrast-enhanced CT or MRI if recurrence is suspected or ultrasonographic evaluation is inconclusive.

## Conclusion

Giant adrenal pseudocysts should be considered in the differential diagnosis of indeterminate upper retroperitoneal cystic lesions, particularly when the ipsilateral adrenal gland is not separately visualized and adjacent organs are markedly displaced. When dense adhesions preclude a safe dissection plane, surgical management should prioritize patient safety and strike an individualized balance between maximal lesion removal and preservation of adjacent organs.

## Data Availability

The original contributions presented in the study are included in the article/Supplementary Material, further inquiries can be directed to the corresponding authors.
